# Behavioral Effects of Systemic, Infralimbic and Prelimbic Injections of a Serotonin 5-HT_2A_ Antagonist in Carioca High- and Low-Conditioned Freezing Rats

**DOI:** 10.3389/fnbeh.2017.00117

**Published:** 2017-07-07

**Authors:** Laura A. León, Vitor Castro-Gomes, Santiago Zárate-Guerrero, Karen Corredor, Antonio P. Mello Cruz, Marcus L. Brandão, Fernando P. Cardenas, J. Landeira-Fernandez

**Affiliations:** ^1^Laboratory of Neuropsychopharmacology, FFCLRP, São Paulo University, Campus USP, and Behavioral Neuroscience Institute (INeC)Ribeirão Preto, São Paulo, Brazil; ^2^Department of Psychology, Pontifical Catholic University of Rio de JaneiroRio de Janeiro, Brazil; ^3^Programa de Psicología, Universidad Sergio ArboledaBogotá, Colombia; ^4^Laboratory of Experimental and Computational Neuroscience, Department of Bio-systems Engineering, Federal University of São João del ReiSão João del Rei, Brazil; ^5^Laboratorio de Neurociencia y Comportamiento, Universidad de los AndesBogotá, Colombia; ^6^Institute of Psychology, University of BrasiliaBrasilia, Brazil

**Keywords:** breeding lines, freezing, contextual fear conditioning, elevated plus maze, serotonin, 5-HT_2A_ receptors, medial prefrontal cortex

## Abstract

The role of serotonin (5-hydroxytryptamine [5-HT]) and 5-HT_2A_ receptors in anxiety has been extensively studied, mostly without considering individual differences in trait anxiety. Our laboratory developed two lines of animals that are bred for high and low freezing responses to contextual cues that are previously associated with footshock (Carioca High-conditioned Freezing [CHF] and Carioca Low-conditioned Freezing [CLF]). The present study investigated whether ketanserin, a preferential 5-HT_2A_ receptor blocker, exerts distinct anxiety-like profiles in these two lines of animals. In the first experiment, the animals received a systemic injection of ketanserin and were exposed to the elevated plus maze (EPM). In the second experiment, these two lines of animals received microinjections of ketanserin in the infralimbic (IL) and prelimbic (PL) cortices and were exposed to either the EPM or a contextual fear conditioning paradigm. The two rat lines exhibited bidirectional effects on anxiety-like behavior in the EPM and opposite responses to ketanserin. Both systemic and intra-IL cortex injections of ketanserin exerted anxiolytic-like effects in CHF rats but anxiogenic-like effects in CLF rats. Microinjections of ketanserin in the PL cortex also exerted anxiolytic-like effects in CHF rats but had no effect in CLF rats. These results suggest that the behavioral effects of 5-HT_2A_ receptor antagonism might depend on genetic variability associated with baseline reactions to threatening situations and 5-HT_2A_ receptor expression in the IL and PL cortices.

**Highlights**

- CHF and CLF rats are two bidirectional lines that are based on contextual fear conditioning.
- CHF rats have a more “anxious” phenotype than CLF rats in the EPM.
- The 5-HT_2A_ receptor antagonist ketanserin had opposite behavioral effects in CHF and CLF rats.
- Systemic and IL injections either decreased (CHF) or increased (CLF) anxiety-like behavior.
- PL injections either decreased (CHF) anxiety-like behavior or had no effect (CLF).

## Introduction

Several studies indicate that contextual fear conditioning represents one of the simplest animal models of investigating anticipatory anxiety (Brandão et al., 2008). It involves placing an animal (e.g., a rat) in a novel environment and delivering a brief unsignaled footshock several minutes later. The next day, the animal freezes when it is returned to the same chamber in the absence of footshock (Landeira-Fernandez, 1996). Conditioned freezing is a direct function of shock intensity (Sigmundi et al., 1983) and depends on the association between the contextual cues associated with the experimental chamber and footshock (Landeira-Fernandez et al., 2006). Classic anxiolytic benzodiazepines, such as midazolam and diazepam (Fanselow and Helmstetter, 1988), and non-benzodiazepine anxiolytics, such as the serotonin (5-hydroxytryptamine [5-HT])-_1A_ receptor agonist ipsapirone (Inoue et al., 1996) and 5-HT reuptake inhibitors citalopram and fluvoxamine (Hashimoto et al., 1996), reduced conditioned freezing. Anxiogenic substances, such as the benzodiazepine inverse agonist dimethoxy-β-carboline, induced freezing behavior similarly to fear conditioning (Fanselow et al., 1991).

Two lines of Wistar rats, termed Carioca High-conditioned Freezing and Low-conditioned Freezing (CHF and CLF, respectively), were selectively bred for high and low levels of freezing in response to contextual cues that were previously associated with footshock (Castro-Gomes and Landeira-Fernandez, 2008). The results of our ongoing breeding program have shown a clear divergence of the conditioned freezing phenotype after only three generations (Castro-Gomes and Landeira-Fernandez, 2008). The presence of different levels of anxiety-like behavior, that are characteristic of each line, can be assessed using several behavioral tests, including the elevated plus maze (EPM), the social interaction test and defensive responses that are induced by electrical stimulation of the dorsal periaqueductal gray (Dias et al., 2009; Galvão et al., 2010; Castro-Gomes et al., 2011, 2014; Salviano et al., 2014). These two lines represent an important tool for investigating the anxiogenic/anxiolytic pharmacological profiles of various compounds (Castro-Gomes et al., 2013).

5-HT is an indoleamine that is intimately connected to the neurocircuitry that underlies anxiety (for review see Millan, 2003; Graeff, 2004). It exerts its behavioral and physiological effects by acting at different receptor subtypes that are distributed into seven G-protein-coupled receptor families (Hoyer et al., 2002; Hannon and Hoyer, 2008). The 5-HT_2_ receptor family (5-HT_2A_, 5-HT_2B_ and 5-HT_2C_) has been the focus of much research interest because of its critical role in modulating anxiety-like behavior in animals and humans (Naughton et al., 2000; Graeff, 2002; Wood, 2003; Gordon and Hen, 2004). The predominant effect of 5-HT in this metabotropic receptor family (especially the 5-HT_2A_ subtype) is excitatory and appears to mediate depolarizing effects (Davie et al., 1988; Eison and Mullins, 1996; Hasuo et al., 2002), but it may also have inhibitory activity or even interact with a number of other inhibitory interneurons (Avesar and Gulledge, 2012; Halberstadt, 2015; Wang et al., 2016).

Paradoxically, the effects of 5-HT_2_-acting drugs on anxiety have been highly inconsistent in animals and humans. For example, systemic administration of 5-HT_2A,2C_ agonists exerts both anxiogenic-like (Charney et al., 1987; Lowy and Meltzer, 1988; Bastani et al., 1990; Rodgers et al., 1992; Gibson et al., 1994; Setem et al., 1999; Jones et al., 2002; Bull et al., 2003; Durand et al., 2003) and anxiolytic-like (Ripoll et al., 2006; Hughes et al., 2012) effects. Similarly, 5-HT_2A,2C_ antagonists have been reported to exert anxiolytic-like effects (Critchley and Handley, 1987; Motta et al., 1992; Kennett et al., 1994; Nic Dhonnchadha et al., 2003), anxiogenic-like effects (Pellow et al., 1987), or no effects (Chaouloff et al., 1997; Setem et al., 1999). Similar inconsistencies have also been reported following direct infusions of 5-HT_2_ agonists and antagonists in anxiety-related postsynaptic brain sites (for review see Menard and Treit, 1999; Graeff, 2002, 2004). For example, 5-HT_2A/2C_ agonist administration in the amygdala (Campbell and Merchant, 2003; de Mello Cruz et al., 2005) and ventral hippocampus (Alves et al., 2004) has been shown to be anxiogenic, whereas microinjections in the dorsal periaqueductal gray have been reported to be anxiolytic (Graeff et al., 1993).

Different experimental procedures, brain sites and selectivity for 5-HT_2_ receptors might contribute to these discrepancies. Moreover, the role of the 5-HT_2_ receptor family in anxiety might depend on genetic variables that are associated with different trait levels of defensive reactions. One of the purposes of the present study was to investigate the effects of systemic injections of a 5-HT_2A_ receptor antagonist in CHF and CLF animals in the EPM. Ketanserin was chosen in this study because of our 25 years’ experience with the use of this drug as a pharmacological tool for the study of the neurobiology of anxiety and fear (Motta et al., 1992; de Luca et al., 2003; Oliveira et al., 2007; Almada et al., 2009). Ketanserin is a 5-HT_2A/2C_ receptor antagonist that has higher affinity for 5-HT_2A_ receptors than 5-HT_2C_ receptors (Kristiansen and Dahl, 1996; López-Giménez et al., 1997; Knight et al., 2004).

EPM is based on rodents’ innate fear of open spaces (Treit et al., 1993). This model has been, behaviorally, physiologically and pharmacologically, validated as an animal model of anxiety in rats (Pellow et al., 1985; Pellow and File, 1986; Reibaud and Böhme, 1993). Factor analyses have also indicated that this test reliably dissociates the anxiety-related effects (open arm entries) from locomotor effects (closed arm entries) of several anxiolytic and anxiogenic agents (File, 1992; Cruz et al., 1994).

The role of 5-HT_2A_ receptors in anxiety might also depend on serotonergic activity in different brain regions. The ventromedial prefrontal cortex (vmPFC) is composed of the infralimbic (IL) and prelimbic (PL) subregions. Serotonergic neurons in both the dorsal and medial raphe nuclei send robust projections to the vmPFC (Azmitia and Segal, 1978; Steinbusch, 1981; Blue et al., 1988). Moreover, 5-HT_2_ receptors, but mainly the 5-HT_2A_ receptor subtype, are widely and densely distributed in both the PL and IL (Pazos et al., 1985; Pompeiano et al., 1994; Santana et al., 2004).

Behavioral results that have been generated with different animal models of anxiety indicate that the IL and PL play distinct and complex roles in conditioned and innate defensive reactions. The IL appears to inhibit the expression of anxiety-like behavior, whereas the PL facilitates its expression through descending projection to the basolateral complex of the amygdala (Likhtik et al., 2014). For example, stimulation of the IL reduced the expression of auditory fear conditioning (Vidal-Gonzalez et al., 2006) but produced anxiety-like behavior in the EPM (Bi et al., 2013). Moreover, inhibition of the IL impaired the acquisition, consolidation and expression of conditioned fear extinction (Sierra-Mercado et al., 2006, 2011; Corcoran and Quirk, 2007). Inactivation of the IL had an anxiolytic-like effect in the EPM (Bi et al., 2013). Stimulation of the PL increased the occurrence of conditioned fear (Vidal-Gonzalez et al., 2006) and anxiety-like behavior in the EPM (Wang et al., 2015). Inactivation of the PL reduced the expression of conditioned fear (Corcoran and Quirk, 2007) and anxiety-like behavior in the EPM (Wang et al., 2015), but PL blockade did not have any effects on innate fear reactions (Corcoran and Quirk, 2007). Neither stimulation nor inactivation of the PL caused any changes in the extinction of contextual (Laurent and Westbrook, 2009) or auditory (Sierra-Mercado et al., 2011) fear conditioning.

These results suggest that the IL and PL play opposite roles in fear conditioning that might depend on nature (i.e., innate or learned) of the threatening stimulus (Lisboa et al., 2010). Therefore, considering the nature of anxiety as well as the presence of 5-HT_2A_ receptors in the PL and IL cortices, the present study also compared in two other experiments the effects of microinjections of the preferential 5-HT_2A_ receptor antagonist ketanserin in the IL and PL in CHF and CLF rats in both innate (EPM) and learned (contextual fear conditioning) models of anxiety.

## Materials and Methods

### Materials

#### Animals

The animals that were used in the present study were selectively bred for high (CHF) and low (CLF) contextual fear conditioning according to procedures described in our previous work (Castro-Gomes and Landeira-Fernandez, 2008). Briefly, albino Wistar rats were selectively bred for differences in defensive freezing behavior in a contextual fear-conditioning paradigm. This protocol involved one acquisition session and one test session. During acquisition, each animal was placed in the observation chamber for 8 min. At the end of this period, three unsignaled 0.5 mA, 1 s electric footshocks were delivered with an intershock interval of 20 s. Three minutes after the last footshock, the animal was returned to its home cage. The test session was conducted approximately 24 h after training. This test consisted of placing the animal for 8 min in the same chamber where the three footshocks were delivered the previous day. No footshock or other stimulation occurred during this period. All of the animals were phenotyped before beginning each experiment. The phenotyping procedure consisted of evaluating the amount of conditioned freezing during the test session (Castro-Gomes and Landeira-Fernandez, 2008). The experiments began 2 months after the phenotyping procedure. The first experiment investigated the effects of systemic intraperitoneal ketanserin administration in CHF and CLF animals of the 10th generation. The second experiment investigated the effects of ketanserin injections in the IL in CHF and CLF animals of the 15th generation. The third experiment investigated the effects of ketanserin injections in the PL in CHF and CLF animals of the 20th generation. Male rats from both the CHF and CLF lines were 4–6 months old at the beginning of each of the three experiments in the present study.

All the animals were born and maintained in the colony room of the PUC-Rio Psychology Department at controlled room temperature (24 ± 1°C) and a 12 h/12 h light/dark cycle (lights on 7:00 AM–7:00 PM). They were housed in groups of 3–5 according to their respective lines in polycarbonate cages (18 cm × 31 cm × 38 cm) with food and water available *ad libitum*. All of the behavioral experiments were conducted during the light phase of the light/dark cycle. The animals were handled once daily for 2 min for 5 days before the beginning of each experiment. The experimental procedures were performed in accordance with the guidelines for experimental animal research that were established by the Brazilian Society of Neuroscience and Behavior (SBNeC) and National Institutes of Health *Guide for the Care and Use of Laboratory Animals*. Animal handling and the methods of sacrifice were reviewed and approved by the Committee for Animal Care and Use of PUC-Rio (protocol no. 20/2009).

#### Apparatus

Contextual fear conditioning was performed in four observational chambers (25 cm × 20 cm × 20 cm) inside sound-attenuating boxes. A video camera was mounted on the back of each observational chamber so that the animal’s behavior could be observed on a monitor in an adjacent room. Background noise (78 dB) was supplied by a white-noise generator. The chamber had a grid floor (15 stainless steel rods spaced 1.5 cm apart) connected to a shock generator (0.5 mA, 1 s duration) and scrambler (AVS, SCR04; São Paulo, Brazil). An interface with four channels (Insight, Ribeirão Preto, Brazil) connected the shock generator to a computer, which allowed the experimenter to apply an electric footshock. A digital multimeter was used to calibrate the shock intensities before each experiment. A 5% ammonium hydroxide solution was used to clean the chamber before and after each test.

The EPM was elevated 50 cm above the floor and had two open arms (50 cm × 10 cm, with 1 cm high edges) and two closed arms (50 cm × 10 cm, with 40 cm high walls) arranged so that arms of the same type were opposite each other. All of the arms were connected by an open central area (10 cm × 10 cm). The tests were performed in a room that was illuminated by a 100-W light bulb that was suspended 1.75 m above the central part of the maze. A 20% alcohol solution was used to clean the maze between trials.

#### Drug

Ketanserin tartrate 97% (Sigma-Aldrich, St. Louis, MO, USA) was dissolved in 2% dimethylsulfoxide (DMSO) vehicle solution. Both the systemic (0.5 mg/kg) and central (5 nmol/μl) doses were selected according to our previous work (Motta et al., 1992; de Luca et al., 2003; Oliveira et al., 2007). Rats in the control group received 2% DMSO dissolved in saline (vehicle).

### Stereotaxic Surgery and Histology

The rats in Experiments 2 and 3 were implanted with bilateral cannulae in the IL or PL. The rats were anesthetized with an intraperitoneal injection of 75 mg/kg ketamine hydrochloride (ketamine 50®, Holliday-Scott SA) and 10 mg/kg xylazine (Seton®, Calier) and mounted in a stereotaxic instrument with the incisor bar set 3.3 mm below the interaural line. Each rat was bilaterally implanted with stainless steel guide cannulae (outer diameter, 0.7 mm) aimed 0.5 mm above the target area. With bregma as the reference for each plane (Paxinos and Watson, 1986), the coordinates for IL cannula implantation were the following: anterior/posterior, 3.2 mm; lateral, ± 0.4 mm; depth, 4.2 mm. The coordinates for PL cannula implantation were the following: anterior/posterior, 3.2 mm; lateral, ± 0.5 mm; depth, 2.4 mm. The guide cannulae were anchored to the skull by dental acrylic and one stainless steel screw. After implantation, the guide cannulae were sealed with a stainless steel wire to prevent blockage. Immediately after cannula implantation, the animals received 0.1 ml of a combination of antibiotics (benzylpenicillin benzathine, 600,000 UI; benzylpenicillin procaine, 300,000 UI; potassium benzylpenicillin, 300,000 UI; dihydrostreptomycin sulfate, 250 mg; streptomycin sulfate, 250 mg, intramuscular) and an analgesic (flunixin, 50 mg/kg, subcutaneous) to prevent infections and decrease post-surgical pain.

At the end of the experiments, the animals were bilaterally injected with 2 μl of 1% Evan’s Blue dye in the IL and PL to verify the accuracy of the injections. The animals were then sacrificed with an overdose of urethane (1.25 g/kg, intraperitoneal; Sigma-Aldrich, St. Louis, MO, USA) and intracardially perfused with 0.9% saline and 4% formalin through the left ventricle. The brains were removed and stored in 10% formalin for at least 2 weeks and then sectioned using a cryostat (CM-1900, Leica, Germany) into 60 μm sections. The injection sites were identified using the rat brain atlas of Paxinos and Watson (1986). Only rats with bilateral cannula sites in the IL or PL were considered for the statistical analysis.

### Methods

#### Experiment 1

The CHF and CLF animals were randomly assigned to the ketanserin (0.5 mg/kg) or vehicle group. Thirty minutes after the intraperitoneal injection, each animal was placed in the center of the EPM facing one of the closed arms. The experimental session lasted 5 min. A highly trained observer who remained blind to the treatment conditions recorded the number of entries into and time spent on the open and closed arms with the aid of computer software. The percentage of open arm entries (100 × open arm entries total arm entries) and percentage of time spent on the open arms (100 × time open/[time open + time closed]) were calculated for each animal as indices of anxiety-like behavior. Based on factor analysis of the rats’ behavior in the EPM (File, 1992; Cruz et al., 1994), the absolute number of closed arm entries was interpreted as a reliable index of general locomotor activity.

#### Experiments 2 and 3

One week after surgery, CHF and CLF animals were randomly assigned to the ketanserin (5 nmol/μl) or vehicle group. Experiment 2 investigated the behavioral effects of ketanserin microinjections in the IL. Experiment 3 investigated the behavioral effects of ketanserin microinjections in the PL. Bilateral infusions were performed through an internal cannula (outer diameter, 0.3 mm) that extended 0.5 mm beyond the guide cannula tip. The cannula was attached to a 10 μl Hamilton syringe via polyethylene-10 tubing. Confirmation of a successful infusion was achieved by monitoring the movement of a small air bubble inside the polyethylene-10 tubing. A volume of 0.5 μl/side was delivered over approximately 2 min through the 10 μl Hamilton syringe, driven by a Harvard syringe pump. Following the infusion, the internal cannula was left in place for an additional 2 min to minimize reflux up the cannula shaft. Previous results from our laboratory demonstrated the effectiveness of these parameters when ketanserin was injected locally into brain structures (Motta et al., 1992; de Luca et al., 2003; Oliveira et al., 2007; Almada et al., 2009). Other results suggest that the drug may diffuse approximately 0.5–1.0 mm from the tip of the infusion cannula (Allen et al., 2008).

Five minutes after the injection, each animal was tested in the EPM. At the end of the EPM test, each animal was returned to the same chamber where contextual fear conditioning occurred to test the duration of freezing as a measure of the conditioned fear response. The animal stayed there for 8 min with no footshock or other stimulation. A time-sampling procedure was used to assess fear conditioning in response to contextual cues. Every 2 s, a well-trained observer recorded episodes of freezing, which were defined as the total absence of movement of the body or vibrissa, with the exception of movement required for respiration.

### Statistical Analysis

The results were statistically analyzed by two-way analysis of variance (ANOVA) to detect overall differences. One independent factor was treatment (ketanserin and vehicle), and the other independent factor was rat line (CLF and CHF). Tukey’s Honestly Significant Difference test was used for *post hoc* pairwise comparisons between groups. In all cases, values of *p* < 0.05 were considered statistically significant. All analysis were performed with SPSS software.

## Results

### Systemic Injection of Ketanserin Induced an Anxiolytic-Like Effect in CHF Animals and Anxiogenic-Like Effect in CLF Animals in the Elevated Plus Maze

**Figure 1:**
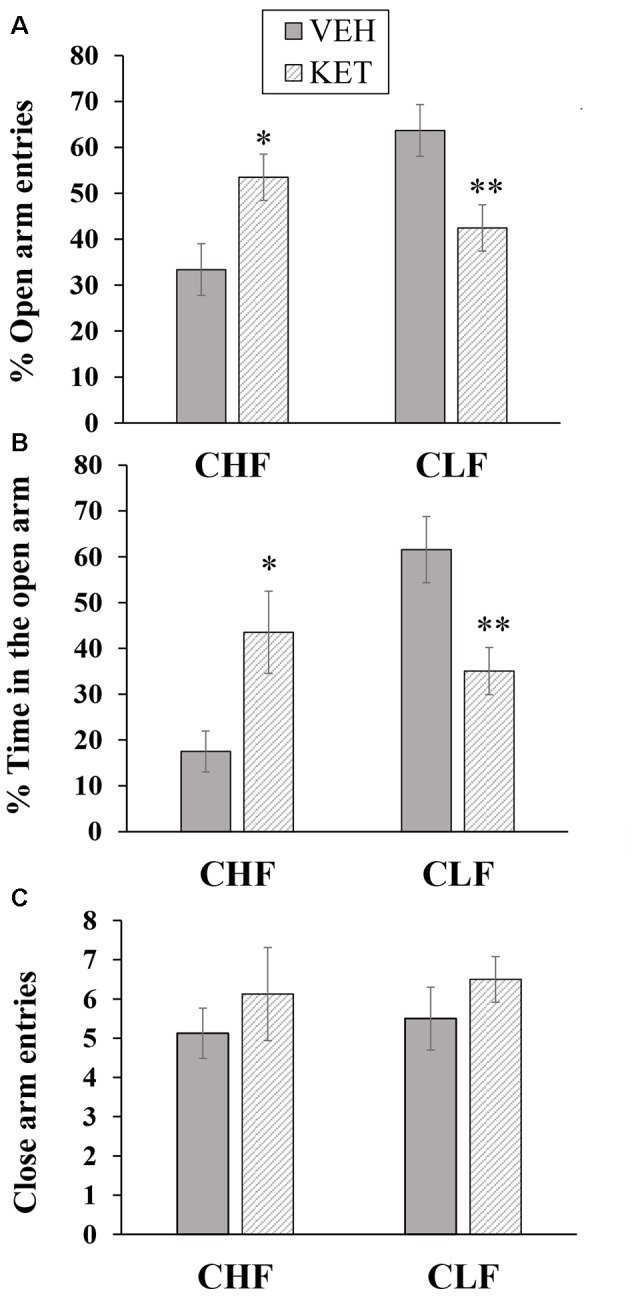
Mean + SEM percentage of open arm entries **(A)**, percent time spent on the open arms **(B)** and closed arm entries **(C)** in the elevated plus maze (EPM) in Carioca High-conditioned Freezing (CHF) and Carioca Low-conditioned Freezing (CLF) animals that received systemic ketanserin (KET) or vehicle (VEH) injections. *p < 0.05 KET vs. VEH among CHF animals; **p < 0.05 KET vs. VEH among CLF animals.

The number of animals in each experimental condition in this experiment was the following: CLF animals injected with vehicle (*n* = 8), CLF animals injected with ketanserin (*n* = 10), CHF animals injected with vehicle (*n* = 8), and CHF animals injected with ketanserin (*n* = 8). Figure 1 shows the behavioral effects in CHF and CLF that received systemic injections of ketanserin or vehicle in the EPM. The two-way ANOVA of the percentage of open arm entries (Figure 1A) indicated a main effect of rat line (*F*_(1,30)_ = 4.71, *p* < 0.05) and a rat line × treatment interaction (*F*_(1,30)_ = 21.66, *p* < 0.01). No main effect of treatment was found (*F*_(1,30)_ = 0.01, *p* > 0.90). The *post hoc* comparisons revealed that systemic ketanserin administration significantly increased the percentage of open arm entries in CHF animals compared with the vehicle but significantly decreased this measure in CLF animals (both *p* < 0.05).

A similar pattern was observed for the percentage of time spent in the open arms (Figure 1B). The two-way ANOVA revealed a main effect of rat line (*F*_(1,30)_ = 7.31, *p* < 0.05) and a rat line × treatment interaction (*F*_(1,30)_ = 15.91, *p* < 0.01). No main effect of treatment was found (*F*_(1,30)_ = 0.01, *p* > 0.90). The *post hoc* comparisons revealed that systemic ketanserin administration significantly increased the percentage of time spent on the open arms in CHF animals compared with vehicle but significantly decreased this measure in CLF animals (both *p* < 0.05).

Figure 1C shows the effects of ketanserin and vehicle on the absolute number of closed arm entries in CHF and CLF animals. No differences were observed among groups. The two-way ANOVA revealed no main effect of rat line or treatment and no interaction between factors (all *p* > 0.05).

### Intra-IL Injection of Ketanserin Induced an Anxiolytic-Like Effect in CHF Animals and Anxiogenic-Like Effect in CLF Animals in Both the Elevated Plus Maze and Contextual Fear Conditioning Paradigm

#### Histology

**Figure 2:**
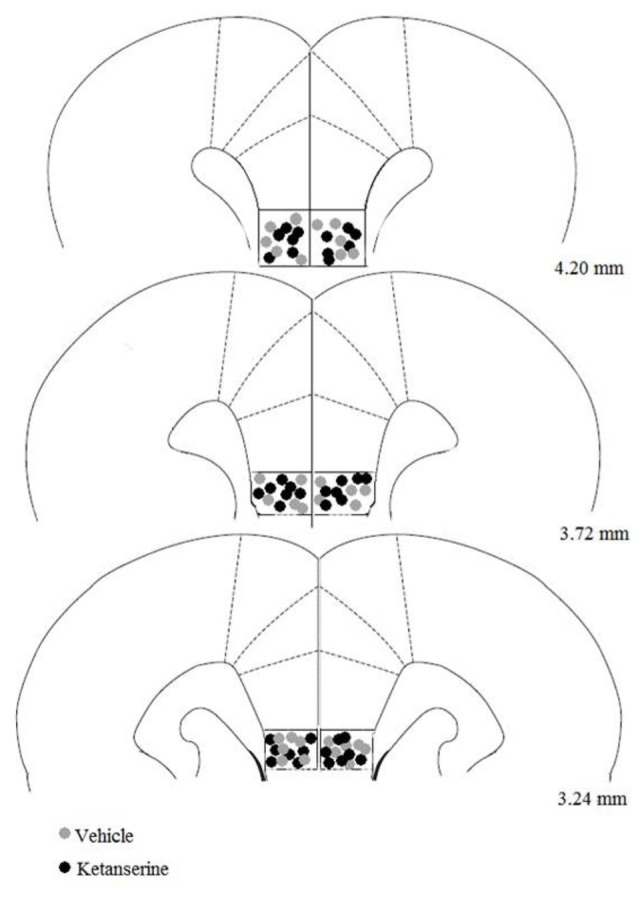
Site of microinjection tips in the infralimbic (IL) cortex. Gray square indicates site of vehicle injection. Black circle indicates site of ketanserin injections. Plates are taken from Paxinos and Watson (1986) and the numbers on the right side of each plate indicate the distance (in millimeters) from bregma.

The histological analysis of the cannula placements confirmed that the infusions were made in the IL region in all animals that were included in the statistical analysis. Four of forty rats in the experiment were excluded because their cannula missed the IL. Figure 2 shows the bilateral microinjection sites in the IL. The final sample size for each group was the following: CLF animals injected with vehicle (*n* = 9), CLF animals injected with ketanserin (*n* = 11), CHF animals injected with vehicle (*n* = 7), and CHF animals injected with ketanserin (*n* = 9).

#### Elevated Plus Maze

**Figure 3:**
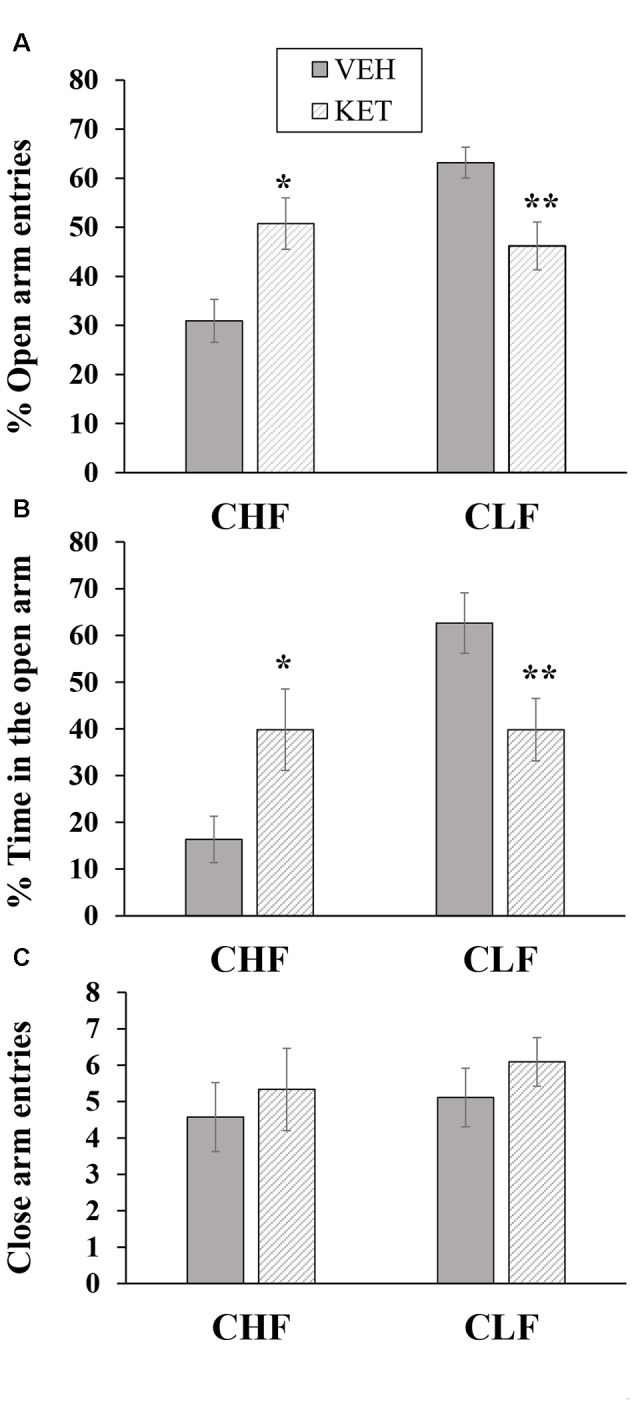
Mean + SEM percentage of open arm entries **(A)**, percent time spent on the open arms **(B)** and closed arm entries **(C)** in the EPM in CHF and CLF animals that received ketanserin (KET) or vehicle (VEH) microinjections in the IL cortex. *p < 0.05 KET vs. VEH among CHF animals; **p < 0.05 KET vs. VEH among CLF animals.

Figure 3 shows the mean ± SEM percentage of open arm entries, percent time spent on the open arms and closed arm entries in the EPM in CHF and CLF animals that received ketanserin or vehicle microinjections in the IL.

The two-way ANOVA of the percentage of open arm entries (Figure 3A) indicated a main effect of rat line (*F*_(1,32)_ = 8.81, *p* < 0.05) and a rat line × treatment interaction (*F*_(1,32)_ = 15.61, *p* < 0.01). No main effect of treatment was found (*F*_(1,32)_ = 0.09, *p* > 0.76). The *post hoc* comparisons revealed that ketanserin microinjections in the IL significantly increased the percentage of open arm entries in CHF animals compared with the vehicle but significantly decreased this measure in CLF animals (both *p* < 0.05).

The two-way ANOVA of the percentage of time spent on the open arms (Figure 3B) revealed a main effect of rat line (*F*_(1,32)_ = 10.41, *p* < 0.05) and a rat line × treatment interaction (*F*_(1,32)_ = 10.41, *p* < 0.05). No main effect of treatment was found (*F*_(1,32)_ = 0.01, *p* > 0.90). The *post hoc* comparisons revealed that ketanserin significantly increased the percentage of time spent on the open arms in CHF animals compared with the vehicle but significantly decreased this measure in CLF animals (both *p* < 0.05).

The two-way ANOVA of the absolute number of closed arm entries in CHF and CLF animals that received microinjections of ketanserin or vehicle (Figure 3C) revealed no main effect of rat line or treatment and no interaction between these factors (all *p* > 0.05).

#### Contextual Fear Conditioning

**Figure 4:**
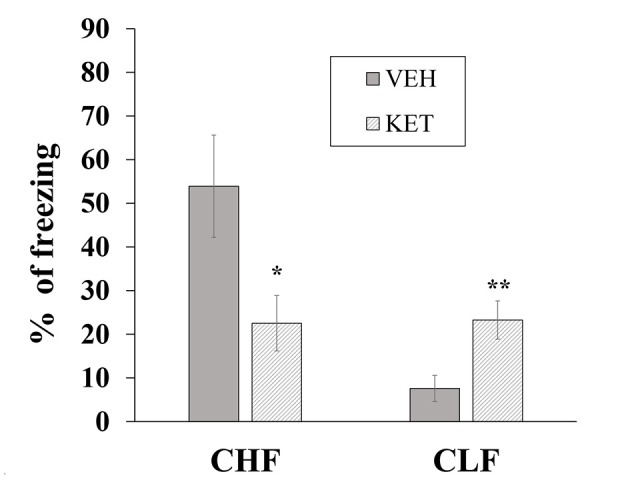
Mean + SEM percentage of time spent freezing in CHF and CLF animals that received ketanserin (KET) or vehicle (VEH) microinjections in the IL cortex. *p < 0.05 KET vs. VEH among CHF animals; **p < 0.05 KET vs. VEH among CLF animals.

Figure 4 shows the mean ± SEM percentage of time spent freezing in CHF and CLF animals that received microinjections of ketanserin or vehicle in the IL. The two-way ANOVA indicated main effects of rat line (*F*_(1,32)_ = 69.12, *p* < 0.01), treatment (*F*_(1,32)_ = 70.61, *p* < 0.01) and a significant rat line × treatment interaction (*F*_(1,32)_ = 66.15, *p* < 0.01). The *post hoc* comparisons indicated that ketanserin microinjections in the IL significantly decreased the percentage of time spent freezing in CHF animals compared with the vehicle but significantly increased this measure in CLF animals (both *p* < 0.05).

### Intra-PL Injection of Ketanserin Induced an Anxiolytic-Like Effect in CHF Animals but no Effect in CLF Animals in Both the Elevated Plus Maze and Contextual Fear Conditioning Paradigm

#### Histology

**Figure 5:**
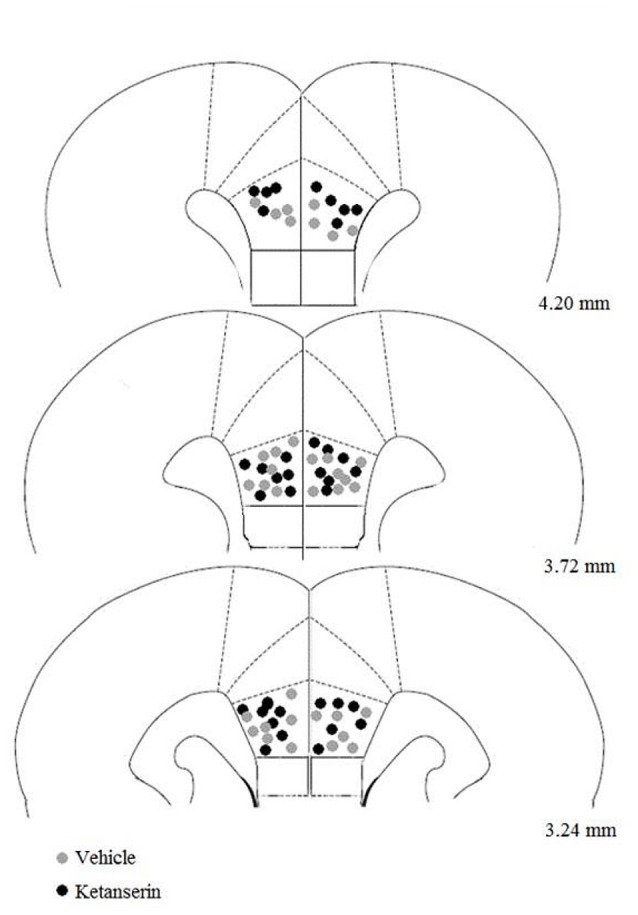
Site of microinjection tips in the prelimbic (PL) cortex. Gray square indicates site of vehicle injection. Black circle indicates site of ketanserin injections. Plates are taken from Paxinos and Watson (1986) and the numbers on the right side of each plate indicate the distance (in millimeters) from bregma.

Figure 5 shows coronal sections of the injection sites in the PL. The injections were distributed throughout the entire rostral-caudal extent of the target area within the PL. Three of thirty-nine rats in the experiment were excluded because their cannula missed the PL. The final sample size for each group was the following: CLF animals injected with vehicle (*n* = 10), CLF animals injected with ketanserin (*n* = 9), CHF animals injected with vehicle (*n* = 8) and CHF animals injected with ketanserin (*n* = 9).

#### Elevated Plus Maze

**Figure 6:**
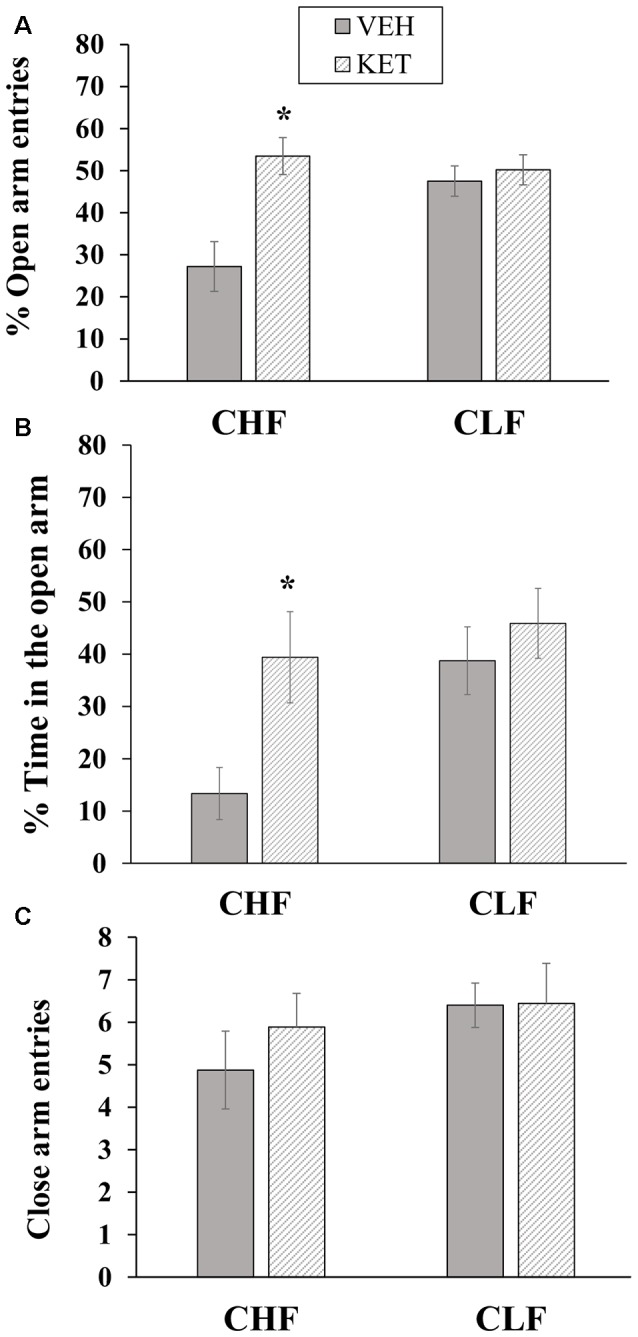
Mean + SEM percentage of open arm entries **(A)**, percent time spent on the open arms **(B)** and closed arm entries **(C)** in the EPM in CHF and CLF animals that received ketanserin (KET) or vehicle (VEH) microinjections in the PL cortex. *p < 0.05 KET vs. VEH among CHF animals.

Figure 6 shows the mean ± SEM percentage of open arm entries, percent time spent on the open arms and closed arm entries in the EPM in CHF and CLF animals that received microinjections of ketanserin or vehicle in the PL.

The two-way ANOVA of the percentage of open arm entries (Figure 6A) revealed a main effect of treatment (*F*_(1,32)_ = 11.01, *p* < 0.05) and a rat line × treatment interaction (*F*_(1,32)_ = 7.32, *p* < 0.01). No main effect of treatment was found (*F*_(1,32)_ = 3.81, *p* > 0.05). The *post hoc* comparisons revealed that ketanserin microinjections in the PL significantly increased the percentage of open arm entries in CHF animals compared with vehicle (*p* < 0.05) but had no effect on this measure in CLF animals (*p* > 0.05).

The two-way ANOVA of the percentage of time spent on the open arms (Figure 6B) revealed main effects of treatment (*F*_(1,32)_ = 6.47, *p* < 0.05) and rat line (*F*_(1,32)_ = 5.98, *p* < 0.05). No interaction between rat line and treatment was found (*F*_(1,32)_ = 2.10, *p* > 0.15).

Figure 6C shows the absolute number of closed arm entries in the EPM maze in CHF and CLF animals that received microinjections of ketanserin or vehicle. The two-way ANOVA revealed no main effect of rat line or treatment and no interaction between these factors (all *p* > 0.05).

#### Contextual Fear Conditioning

**Figure 7:**
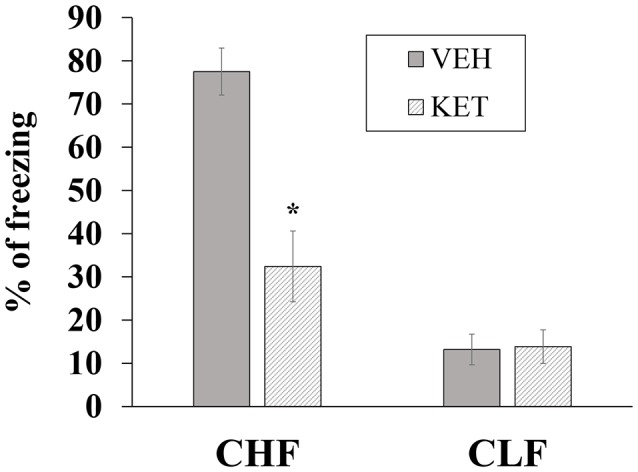
Mean + SEM percentage of time spent freezing in CHF and CLF animals that received ketanserin (KET) or vehicle (VEH) microinjections in the PL cortex. *p < 0.05 KET vs. VEH among CHF animals.

Figure 7 shows the mean ± SEM percentage of time spent freezing in CHF and CLF animals that received microinjections of ketanserin or vehicle in the PL. The two-way ANOVA indicated main effects of rat line (*F*_(1,32)_ = 26.02, *p* < 0.01), treatment (*F*_(1,32)_ = 27.30, *p* < 0.01) and a significant rat line × treatment interaction (*F*_(1,32)_ = 28.42, *p* < 0.01). The *post hoc* comparisons indicated that ketanserin microinjections in the PL significantly decreased conditioned freezing in CHF animals compared with vehicle (*p* < 0.05) but had no effect in CLF animals (*p* > 0.05).

## Discussion

Although there are several animal models of anxiety, very few have highlighted individual differences in the vulnerability to threatening situations as an important variable for studying behavioral responses to pharmacological compounds (Steimer, 2011; Beckers et al., 2013). Selectively bred models of trait anxiety represent an important tool for investigating neural pathophysiological mechanisms associated with anxiety disorders (Castro-Gomes et al., 2013). The present study evaluated the behavioral response to the preferential 5-HT_2A_ receptor antagonist ketanserin in two lines of animals that were selectively bred for high (CHF) and low (CLF) freezing responses to contextual cues that were previously associated with footshock.

The present results from these two lines of animals from the 10th, 15th and 20th generations consistently showed that CHF rats displayed a significantly more “anxious” phenotype, reflected by open arm parameters in the EPM compared with CLF animals. No differences were found between CHF and CLF rats in the number of closed arm entries, suggesting that the anxiety-like profile of CHF animals was not attributable to locomotor impairment but rather to increases in aversion to the open arms. These behavioral differences between CHF and CLF rats in the EPM are consistent with previous studies from our laboratory and indicate that the conditioned freezing parameter that is used for our breeding program remained stable in a different threatening situation across different generations (Hassan et al., 2013, 2015; Dias et al., 2014; Mousovich-Neto et al., 2015).

Importantly, a biological phenomenon known as genetic drift (Falconer and Mackay, 1996) might represent a confounding factor in our breeding program, in which allele frequencies significantly increase or decrease, possibly leading to differential fixation of the alleles in the CHF and CFL lines as a result of the small size of the two populations. Nevertheless, the present results and past findings from our group strongly indicate that our artificial selection program has indeed resulted in contrasting phenotypes between our two lines that are not attributable to random genetic effects, which cannot be entirely excluded in any evolutionary process that involves small breeding groups.

Systemic injections of ketanserin induced opposite effects in these two lines of rats when the animals were evaluated in the same animal model of anxiety. Ketanserin administration in CHF animals increased both the percentage of entries into and time spent on the open arms of the EPM, without changing the absolute number of closed arm entries. This indicates a selective anxiolytic-like effect without locomotor interference in this test. Conversely, CLF animals that received systemic ketanserin injections exhibited a behavioral pattern suggestive of an anxiogenic-like action in the EPM, reflected by a decrease in open arm exploration and no changes in closed arm entries. These results are consistent with a previous study that reported a bidirectional effect of systemic ketanserin administration in the EPM in female rats with different basal levels of anxiety associated with hormonal states (Díaz-Véliz et al., 1997). Ketanserin produced an anxiogenic-like effect in low-anxiety females that had high estrogen levels (proestrus) but produced an anxiolytic-like effect in high-anxiety females that had low estrogen levels (diestrus). León et al. (2009) showed that methylenedioxymethamphetamine and fluoxetine administration also had opposite effects, depending on whether the subjects were pre-exposed to chronic mild stress, suggesting differential effects of the drug that depended on basal conditions (León et al., 2009). Therefore, differences in baseline levels of anxiety appear to be important for determining the behavioral effects of serotonergic manipulations in the EPM. We previously reported in the rat EPM that the anxiogenic-like effects of the 5-HT_2_ receptor agonist TFMPP administered systemically were prevented by intra-amygdala infusions of the mixed 5-HT_2A/2C_-receptor antagonist ritanserin, which does not affect basal levels of anxiety in this animal model (de Mello Cruz et al., 2005).

The behavioral effects of pharmacological interventions are a dynamic process that involves both environmental and genetic factors. The present results highlight the importance of considering the underlying heterogeneity of a given experimental animal population. This is particularly important in basic research that investigates the role of serotonergic activity in anxiety. Conflicting results might be found because most experiments use very heterogeneous populations with considerable variations in anxiolytic-like responses to the same threatening situation. Ignoring the impact of individual differences in behavioral pharmacology research that is performed with heterogeneous populations of animals might mask behavioral effects due to an average result across different levels of defensive responses that these animals might present to cope with a threatening situation (Veenema et al., 2004; Beerling et al., 2011; Castro et al., 2012; Duclot and Kabbaj, 2013). Human and animal studies have shown individual differences in ways of coping with environmental challenges (Blanchard et al., 2001; de Kloet et al., 2005; Bardi et al., 2012; Metna-Laurent et al., 2012; Coppens et al., 2013). Using similar populations, some individuals display higher vulnerability to the development of anxiety-related disorders when faced with threatening situations, whereas other individuals seem to be more resilient to the development these types of pathologies (Bolger and Zuckerman, 1995; Hammen, 2005; Aisa et al., 2008; Uchida et al., 2008; Sandi and Richter-Levin, 2009; Oitzl et al., 2010). Therefore, individual variability needs to be taken into account in animal models of anxiety. Using animal lines that express an anxious-like phenotype is an important methodology for investigating the ways in which the underlying neuropharmacology might contribute to the observed behavioral differences.

The present study also investigated the participation of 5-HT_2_ receptors in the IL and the PL subregions of the vmPFC in CHF and CLF animals in the EPM and contextual fear conditioning paradigm. Intra-IL acute infusions of ketanserin induced the same bidirectional behavioral effects in the EPM as systemic injections (i.e., an anxiolytic-like effect in CHF rats but an anxiogenic-like effect in CLF rats), and these effects extended to contextual fear conditioning. Intra-PL acute infusions of ketanserin also had an anxiolytic-like effect in CHF animals but no effect in CLF rats in both models of anxiety. These results suggest that participation of the IL and PL on anxiety-like behavior might not depend solely on the nature (i.e., innate or learned) of the threatening stimulus, as suggested by Corcoran and Quirk (2007) and Sierra-Mercado et al. (2006), but rather on the genetic vulnerability of specific animals to the threatening situation. Moreover, ketanserin injections in either the IL or PL produced the same anxiolytic-like effect in CHF animals. Behavioral differences between ketanserin injections in the IL and PL were only observed in CLF animals, in which an anxiogenic-like effect was only observed when it was injected in the IL and not in the PL.

The IL sends descending projection to the basolateral complex of the amygdala (Likhtik et al., 2014). These projections seem to play an important role in the extinction of conditioned fear (McDonald, 1998; Vertes, 2004). Inactivation of the IL impaired the consolidation and retrieval of extinction of fear conditioning (Quirk et al., 2000; Laurent and Westbrook, 2009). The present study only found an anxiogenic-like effect in CLF rats when the IL region was microinjected with ketanserin. Surprisingly, CHF animals displayed an anxiolytic-like response to microinjection of the same 5-HT_2_ antagonist. This could be related to differences in 5-HT_2_ receptor expression in this subregion of the vmPFC in both lines of animals. 5-HT_2_ receptor downregulation after chronic treatment with antidepressants (Peroutka and Snyder, 1980) and serotonin receptor agonists has been reported (Conn and Sanders-Bush, 1986; Leysen et al., 1987a,b; Smith et al., 1999).

Neurons in the PL play an excitatory role in conditioned fear behavior. This excitatory effect appears also to be mediated by descending projections to the basolateral complex of the amygdala (Likhtik et al., 2014). Previous results indicated that pharmacological inhibition of the PL reduced the expression of conditioned fear (Corcoran and Quirk, 2007). Furthermore, immunohistochemistry indicated that the PL exhibits greater activation when animals are reexposed to contextual cues that were previously associated with footshock (Lemos et al., 2010). Our results in CHF animals are consistent with these previous studies and highlight the participation of postsynaptic 5-HT_2A_ receptors in the PL in contextual fear conditioning. However, ketanserin did not induce any reduction in conditioned fear among CLF animals, which might be attributable to a floor effect because the animals that received vehicle injections already had very low levels of the freezing response.

Importantly, the present study has several limitations that need to be considered when interpreting the findings. One of the limitations refers to the selectivity of ketanserin. Although this drug has been extensively used as a pharmacological tool to efficiently block 5-HT_2A_ receptors (Kristiansen and Dahl, 1996; López-Giménez et al., 1997; Knight et al., 2004), it also has high binding affinity for both histamine H_1_ (Wouters et al., 1985; Ghoneim et al., 2006) and α_1_-adrenergic (Hoyer et al., 1987; Israilova et al., 2002) receptors. Thus, the participation of vmPFC 5-HT_2A_ receptors in bidirectional behavioral changes in CHF and CLF rats should not be considered in isolation. Although beyond the scope of the present study, consideration should be given to the possible influence of H_1_ and α_1_-adrenergic receptors in the present results. Future studies should further investigate the involvement of 5-HT_2A_ receptors in the vmPFC in CHF and CLF animals.

## Author Contributions

Substantial contributions to the conception or design of the work; or the acquisition, analysis, or interpretation of data for the work; and drafting the work or revising it critically for important intellectual content; and final approval of the version to be published; and agreement to be accountable for all aspects of the work in ensuring that questions related to the accuracy or integrity of any part of the work are appropriately investigated and resolved. All authors listed, have made substantial, direct and intellectual contribution to the work, and approved it for publication.

## Conflict of Interest Statement

The authors declare that the research was conducted in the absence of any commercial or financial relationships that could be construed as a potential conflict of interest.
